# Supplementary material for: Methane emissions offset atmospheric carbon dioxide uptake in coastal macroalgae, mixed vegetation and sediment ecosystems
Source: Nat Commun. 2023 Jan 3;14:42. doi: 10.1038/s41467-022-35673-9 (PMC9810657; doi:10.1038/s41467-022-35673-9)
Supplement: Supplementary file 1 — Supplementary Information [file 41467_2022_35673_MOESM1_ESM.pdf]

# Supplementary Material for

# Methane emissions offset atmospheric carbon dioxide uptake in coastal macroalgae, mixed vegetation and sediment ecosystems

Florian Roth[1,2]*, Elias Broman[1,3], Xiaole Sun[4,1], Stefano Bonaglia[5], Francisco Nascimento[1,3], John Prytherch[6], Volker Brüchert[7,8], Maysoon Lundevall Zara[7], Märta Brunberg[2], Marc C. Geibel[1], Christoph Humborg[1,2], and Alf Norkko[1,2]

[1] Baltic Sea Centre, Stockholm University, Stockholm, Sweden
[2] Tvärminne Zoological Station, University of Helsinki, Hanko, Finland
[3] Department of Ecology, Environment and Plant Sciences, Stockholm University, Stockholm, Sweden
[4] Center of Deep Sea Research, Institute of Oceanology, Chinese Academy of Sciences, Qingdao, China
[5] Department of Marine Sciences, University of Gothenburg, Gothenburg, Sweden
[6] Department of Meteorology, Stockholm University, Stockholm, Sweden
[7] Department of Geological Sciences, Stockholm University, Stockholm, Sweden
[8] Bolin Centre for Climate Research, Stockholm University, Stockholm, Sweden

*Corresponding author email address: florian.roth@su.se

**Supplementary Table 1.** Daily net sea-air fluxes of $CO_2$, $CH_4$, $CO_2$-eq. of $CH_4$, and the net greenhouse gas (GHG) across four seasons in three coastal ecosystems. Values are means ± standard error. Positive fluxes refer to an efflux from the water to the atmosphere (source), while negative fluxes depict an uptake of atmospheric GHGs (sink). *$CO_2$-equivalent $CH_4$ fluxes were calculated using the sustained-flux global warming potential (SGWP) on a 100-year time horizon of 45 (21). †The net GHG balance is calculated based on net $CO_2$ and net $CO_2$-eq. $CH_4$ fluxes.

| **Habitat** | **Season** | **Net $CO_2$ flux (mg $CO_2$ $m^{-2}$ $d^{-1}$)** | **Net $CH_4$ flux (mg $CH_4$ $m^{-2}$ $d^{-1}$)** | **Net $CO_2$-eq. $CH_4$ flux* (mg $CO_2$-eq. $m^{-2}$ $d^{-1}$)** | **Net GHG balance† (mg $CO_2$-eq. $m^{-2}$ $d^{-1}$)** |
|---|---|---|---|---|---|
| Macroalgae | Spring | -148 ± 14 | 0.2 ± 0.0 | 9 ± 1 | -139 ± 13 |
| | Summer | -385 ± 54 | 1.8 ± 0.1 | 81 ± 6 | -303 ± 51 |
| | Fall | -159 ± 15 | 1.6 ± 0.1 | 70 ± 6 | -89 ± 13 |
| | Winter | 117 ± 15 | 0.1 ± 0.0 | 1 ± 0 | 118 ± 15 |
| Mixed vegetation | Spring | -126 ± 15 | 0.3 ± 0.0 | 16 ± 2 | -111 ± 14 |
| | Summer | -763 ± 99 | 2.7 ± 0.2 | 122 ± 9 | -641 ± 92 |
| | Fall | -275 ± 45 | 2.9 ± 0.3 | 129 ± 14 | -146 ± 34 |
| | Winter | 390 ± 35 | 0.1 ± 0.0 | 4 ± 0 | 394 ± 35 |
| Bare sediments | Spring | -21 ± 8 | 0.1 ± 0.1 | 7 ± 1 | -15 ± 7 |
| | Summer | 157 ± 59 | 1.5 ± 0.1 | 66 ± 4 | 223 ± 59 |
| | Fall | -132 ± 17 | 2.5 ± 0.2 | 112 ± 10 | -21 ± 11 |
| | Winter | 326 ± 33 | 0.1 ± 0.0 | 2 ± 0 | 328 ± 33 |

**Supplementary Table 2.** Results of the Analysis of Variance of Aligned Rank Transformed Data of the daily net $CH_4$ fluxes across habitats and seasons. The ARTool R package was used to implement the Aligned Rank Transform for conducting nonparametric analyses of variance on the factorial model ($CH_4$ ~ Season*Habitat). The ART-C procedure was used to test for differences in pairwise combinations of levels between factors and interactions (i.e., "Contrasts"). P-values of the post hoc test are adjusted with the Tukey method.

| Term | Df | Df.res | Sum Sq | Sum Sq.res | F value | Pr(>F) |
|---|---|---|---|---|---|---|
| Season | 3 | 2098 | 301549223 | 4.13E+08 | 510.79 | **<0.0001** |
| Habitat | 2 | 2098 | 28390870 | 5.94E+08 | 50.13 | **<0.0001** |
| Season:Habitat | 6 | 2098 | 46288491 | 5.85E+08 | 27.66 | **<0.0001** |

| Contrast | Estimate | SE | df | t ratio | p value |
|---|---|---|---|---|---|
| Fall, Macroalgae - Fall, Mixed vegetation | -162.92 | 36.45 | 2098 | -4.47 | **0.0005** |
| Fall, Macroalgae - Fall, Sediment | -138.84 | 37.81 | 2098 | -3.67 | **0.0132** |
| Fall, Macroalgae - Spring, Macroalgae | 640.14 | 38.49 | 2098 | 16.63 | **<0.0001** |
| Fall, Macroalgae - Summer, Macroalgae | -134.72 | 44.88 | 2098 | -3.00 | 0.1086 |
| Fall, Macroalgae - Winter, Macroalgae | 1076.21 | 43.70 | 2098 | 24.63 | **<0.0001** |
| Fall, Mixed vegetation - Fall, Sediment | 24.08 | 38.50 | 2098 | 0.63 | 1.0000 |
| Fall, Mixed vegetation - Spring, Mixed vegetation | 610.34 | 38.26 | 2098 | 15.95 | **<0.0001** |
| Fall, Mixed vegetation - Summer, Mixed vegetation | -211.61 | 52.81 | 2098 | -4.01 | **0.0037** |
| Fall, Mixed vegetation - Winter, Mixed vegetation | 892.01 | 39.62 | 2098 | 22.52 | **<0.0001** |
| Fall, Sediment - Spring, Sediment | 884.90 | 40.33 | 2098 | 21.94 | **<0.0001** |
| Fall, Sediment - Summer, Sediment | -32.22 | 45.42 | 2098 | -0.71 | 0.9999 |
| Fall, Sediment - Winter, Sediment | 1042.39 | 47.05 | 2098 | 22.15 | **<0.0001** |
| Spring, Macroalgae - Spring, Mixed vegetation | -192.72 | 40.21 | 2098 | -4.79 | **0.0001** |
| Spring, Macroalgae - Spring, Sediment | 105.91 | 40.97 | 2098 | 2.59 | 0.2886 |
| Spring, Macroalgae - Winter, Macroalgae | 436.07 | 45.99 | 2098 | 9.48 | **<0.0001** |
| Spring, Mixed vegetation - Spring, Sediment | 298.64 | 40.11 | 2098 | 7.45 | **<0.0001** |
| Spring, Mixed vegetation - Summer, Mixed vegetation | -821.95 | 53.59 | 2098 | -15.34 | **<0.0001** |
| Spring, Mixed vegetation - Winter, Mixed vegetation | 281.67 | 40.65 | 2098 | 6.93 | **<0.0001** |
| Spring, Sediment - Summer, Sediment | -917.12 | 45.90 | 2098 | -19.98 | **<0.0001** |
| Spring, Sediment - Winter, Sediment | 157.50 | 47.51 | 2098 | 3.31 | **0.0437** |
| Summer, Macroalgae - Summer, Mixed vegetation | -239.81 | 58.94 | 2098 | -4.07 | **0.0029** |
| Summer, Macroalgae - Winter, Macroalgae | 1210.93 | 51.45 | 2098 | 23.54 | **<0.0001** |
| Summer, Mixed vegetation - Summer, Sediment | 203.47 | 58.05 | 2098 | 3.51 | **0.0235** |
| Summer, Mixed vegetation - Winter, Mixed vegetation | 1103.62 | 54.56 | 2098 | 20.23 | **<0.0001** |
| Summer, Sediment - Winter, Sediment | 1074.61 | 51.90 | 2098 | 20.70 | **<0.0001** |
| Winter, Macroalgae - Winter, Mixed vegetation | -347.12 | 46.37 | 2098 | -7.49 | **<0.0001** |
| Winter, Macroalgae - Winter, Sediment | -172.66 | 51.90 | 2098 | -3.33 | **0.0422** |
| Winter, Mixed vegetation - Winter, Sediment | 174.46 | 47.97 | 2098 | 3.64 | **0.0149** |

**Supplementary Table 3.** Results of the Analysis of Variance of Aligned Rank Transformed Data of the daily net $CO_2$ fluxes across habitats and seasons. The ARTool R package was used to implement the Aligned Rank Transform for conducting nonparametric analyses of variance on the factorial model ($CO_2$ ~ Season*Habitat). The ART-C procedure was used to test for differences in pairwise combinations of levels between factors and interactions (i.e., "Contrasts"). P-values of the post hoc test are adjusted with the Tukey method.

| Term | Df | Df.res | Sum Sq | Sum Sq.res | F value | Pr(>F) |
|---|---|---|---|---|---|---|
| Season | 3 | 2098 | 235125157 | 521997662 | 315.00 | **<0.0001** |
| Habitat | 2 | 2098 | 51513911 | 657353277 | 82.21 | **<0.0001** |
| Season:Habitat | 6 | 2098 | 54204608 | 671646215 | 28.22 | **<0.0001** |

| Contrast | Estimate | SE | df | t ratio | p value |
|---|---|---|---|---|---|
| Fall, Macroalgae - Fall, Mixed vegetation | -24.04 | 40.07 | 2098 | -0.60 | 1.0000 |
| Fall, Macroalgae - Fall, Sediment | -126.07 | 41.57 | 2098 | -3.03 | 0.0999 |
| Fall, Macroalgae - Spring, Macroalgae | 16.22 | 42.31 | 2098 | 0.38 | 1.0000 |
| Fall, Macroalgae - Summer, Macroalgae | 107.61 | 49.33 | 2098 | 2.18 | 0.5636 |
| Fall, Macroalgae - Winter, Macroalgae | -847.39 | 48.04 | 2098 | -17.64 | **<0.0001** |
| Fall, Mixed vegetation - Fall, Sediment | -102.03 | 42.33 | 2098 | -2.41 | 0.3990 |
| Fall, Mixed vegetation - Spring, Mixed vegetation | -79.69 | 42.06 | 2098 | -1.89 | 0.7629 |
| Fall, Mixed vegetation - Summer, Mixed vegetation | 323.44 | 58.05 | 2098 | 5.57 | **<0.0001** |
| Fall, Mixed vegetation - Winter, Mixed vegetation | -1022.44 | 43.55 | 2098 | -23.48 | **<0.0001** |
| Fall, Sediment - Spring, Sediment | -318.49 | 44.34 | 2098 | -7.18 | **<0.0001** |
| Fall, Sediment - Summer, Sediment | -468.00 | 49.93 | 2098 | -9.37 | **<0.0001** |
| Fall, Sediment - Winter, Sediment | -943.61 | 51.73 | 2098 | -18.24 | **<0.0001** |
| Spring, Macroalgae - Spring, Mixed vegetation | -119.94 | 44.20 | 2098 | -2.71 | 0.2202 |
| Spring, Macroalgae - Spring, Sediment | -460.78 | 45.04 | 2098 | -10.23 | **<0.0001** |
| Spring, Macroalgae - Summer, Macroalgae | 91.39 | 51.79 | 2098 | 1.76 | 0.8370 |
| Spring, Macroalgae - Winter, Macroalgae | -863.61 | 50.56 | 2098 | -17.08 | **<0.0001** |
| Spring, Mixed vegetation - Spring, Sediment | -340.84 | 44.09 | 2098 | -7.73 | **<0.0001** |
| Spring, Mixed vegetation - Summer, Mixed vegetation | 403.12 | 58.91 | 2098 | 6.84 | **<0.0001** |
| Spring, Mixed vegetation - Winter, Mixed vegetation | -942.76 | 44.68 | 2098 | -21.10 | **<0.0001** |
| Spring, Sediment - Summer, Sediment | -149.51 | 50.45 | 2098 | -2.96 | 0.1202 |
| Spring, Sediment - Winter, Sediment | -625.12 | 52.23 | 2098 | -11.97 | **<0.0001** |
| Summer, Macroalgae - Summer, Mixed vegetation | 191.79 | 64.79 | 2098 | 2.96 | 0.1212 |
| Summer, Macroalgae - Summer, Sediment | -701.68 | 56.56 | 2098 | -12.41 | **<0.0001** |
| Summer, Macroalgae - Winter, Macroalgae | -955.00 | 56.56 | 2098 | -16.88 | **<0.0001** |
| Summer, Mixed vegetation - Summer, Sediment | -893.48 | 63.81 | 2098 | -14.00 | **<0.0001** |
| Summer, Mixed vegetation - Winter, Mixed vegetation | -1345.88 | 59.98 | 2098 | -22.44 | **<0.0001** |
| Summer, Sediment - Winter, Sediment | -475.60 | 57.06 | 2098 | -8.34 | **<0.0001** |
| Winter, Macroalgae - Winter, Mixed vegetation | -199.09 | 50.98 | 2098 | -3.91 | **0.0055** |

**Supplementary Table 4.** Measured environmental variables in the three habitats across four seasons. Values include all measurements over 24h cycles for multiple days within the seasonal sampling campaigns (i.e., spring = 18 – 29 May; summer = 06 – 11 July; fall = 22 October – 2 November; and winter = 30 November – 08 December 2020). Accordingly, values are not representative for the "meteorological" seasons at the study locations. Note: Daily-integrated sea-air fluxes of $CO_2$ and $CH_4$ are not based on the mean values presented here, but result from continuous measurements averaged to 15 min intervals.

| | **Spring** | | **Summer** | | **Fall** | | **Winter** | |
|---|---|---|---|---|---|---|---|---|
| | Range | Mean ± SD | Range | Mean ± SD | Range | Mean ± SD | Range | Mean ± SD |
| **Macroalgae** | | | | | | | | |
| Water temperature (°C) | 8.55 – 11.58 | 9.77 ± 0.76 | 17.08 – 22.17 | 19.80 ± 1.53 | 10.82 – 12.59 | 11.56 ± 0.43 | 5.87 – 6.62 | 6.26 ± 0.16 |
| Salinity | 6.17 – 6.66 | 6.62 ± 0.08 | 6.47 – 6.61 | 6.52 ± 0.04 | 6.34 – 6.50 | 6.43 ± 0.06 | 6.84 – 7.03 | 6.99 ± 0.04 |
| $pCO_2$ water (µatm) | 134 – 352 | 235 ± 53 | 62 – 635 | 245 ± 137 | 73 – 537 | 264 ± 119 | 349 – 710 | 620 ± 64 |
| $CH_4$ water (nmol/L) | 17 – 101 | 41 ± 15 | 41 – 260 | 144 ± 53 | 45 – 169 | 80 ± 24 | 9 – 23 | 13 ± 3 |
| Windspeed (m/s) | 0.1 – 9.1 | 2.1 ± 1.6 | 0.1 – 9.4 | 2.7 ± 1.6 | 0.3 – 11.9 | 4.9 ± 2.7 | 0.1 – 7.2 | 1.7 ± 1.3 |
| **Mixed vegetation** | | | | | | | | |
| Water temperature (°C) | 8.43 – 11.32 | 9.53 ± 0.85 | 16.53 – 22.04 | 19.93 ± 1.80 | 10.21 – 12.81 | 11.46 ± 0.75 | 5.69 – 6.90 | 6.54 ± 0.28 |
| Salinity | 6.39 – 6.66 | 6.61 ± 0.05 | 6.46– 6.58 | 6.51 ± 0.04 | 6.36 – 6.49 | 6.41 ± 0.02 | 6.78 – 7.05 | 6.96 ± 0.09 |
| $pCO_2$ water (µatm) | 131 – 765 | 317 ± 112 | 55 – 1628 | 313 ± 286 | 78 – 617 | 206 ± 138 | 505 – 813 | 669 ± 73 |
| $CH_4$ water (nmol/L) | 17 – 103 | 56 ± 17 | 53 – 460 | 186 ± 71 | 58 – 204 | 119 ± 33 | 6 – 37 | 18 ± 7 |
| Windspeed (m/s) | 0.1 – 9.5 | 2.4 ± 1.8 | 0.1 – 12.5 | 2.9 ± 1.8 | 0.6 – 11.3 | 5.1 ± 2.1 | 0.1 – 14.1 | 3.2 ± 2.6 |
| **Bare sediments** | | | | | | | | |
| Water temperature (°C) | 8.39 – 11.39 | 9.51 ± 0.71 | 16.59 – 21.75 | 20.12 ± 1.64 | 10.34 – 12.86 | 11.85 ± 0.40 | 5.73 – 6.45 | 6.13 ± 0.19 |
| Salinity | 6.21 – 6.67 | 6.61 ± 0.07 | 6.47 – 6.57 | 6.51 ± 0.03 | 6.01 – 6.49 | 6.38 ± 0.07 | 6.78 – 7.01 | 6.92 ± 0.11 |
| $pCO_2$ water (µatm) | 238 – 735 | 414 ± 85 | 122 – 1245 | 379 ± 155 | 110 – 759 | 465 ± 201 | 552 – 756 | 604 ± 35 |
| $CH_4$ water (nmol/L) | 20 – 69 | 40 ± 8 | 41 – 324 | 160 ± 52 | 34 – 115 | 69 ± 17 | 6 – 17 | 9 ± 2 |
| Windspeed (m/s) | 0.1 – 7.4 | 1.7 ± 1.5 | 0.1 – 9.8 | 3.3 ± 2.0 | 0.9 – 9.6 | 4.8 ± 1.6 | 0.2 – 13.4 | 3.4 ± 2.0 |

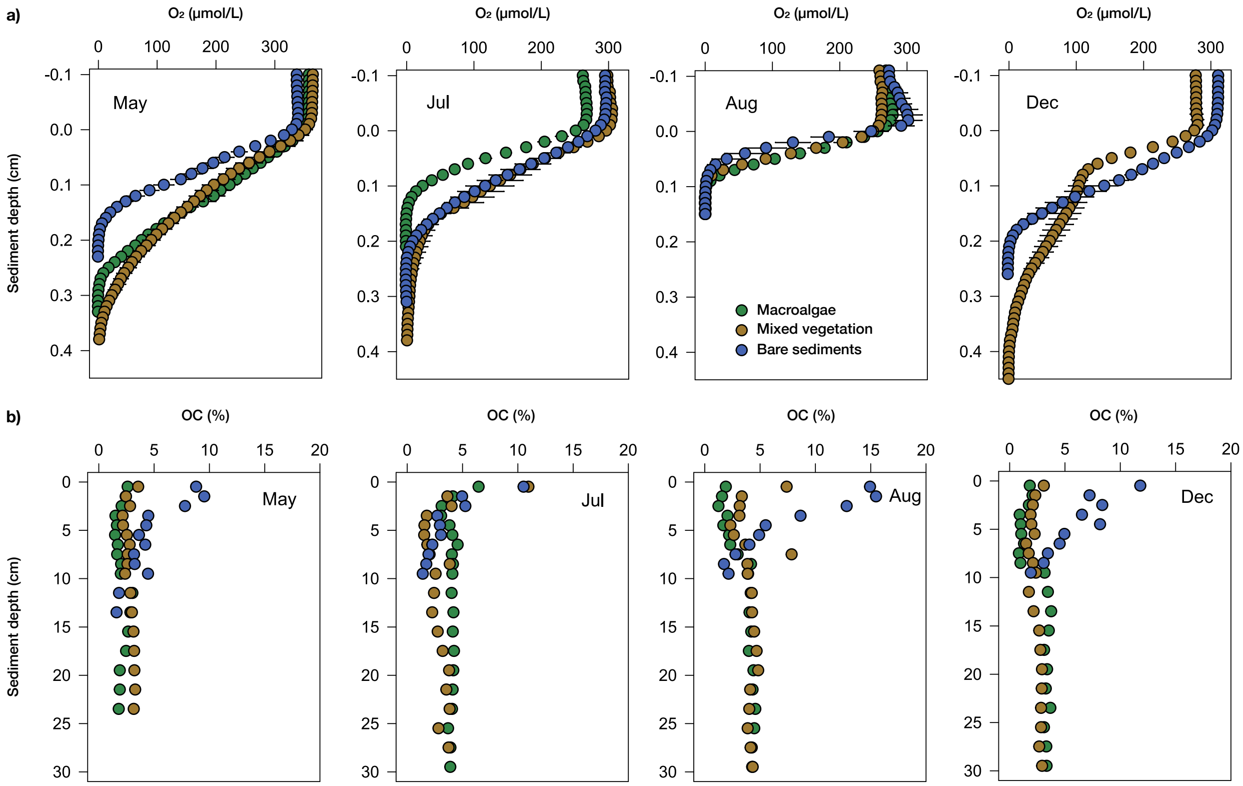


**Supplementary Figure 1.** Seasonal sediment microprofiles for $O_2$ (a) and organic carbon (OC) contents (b) in the three habitats.

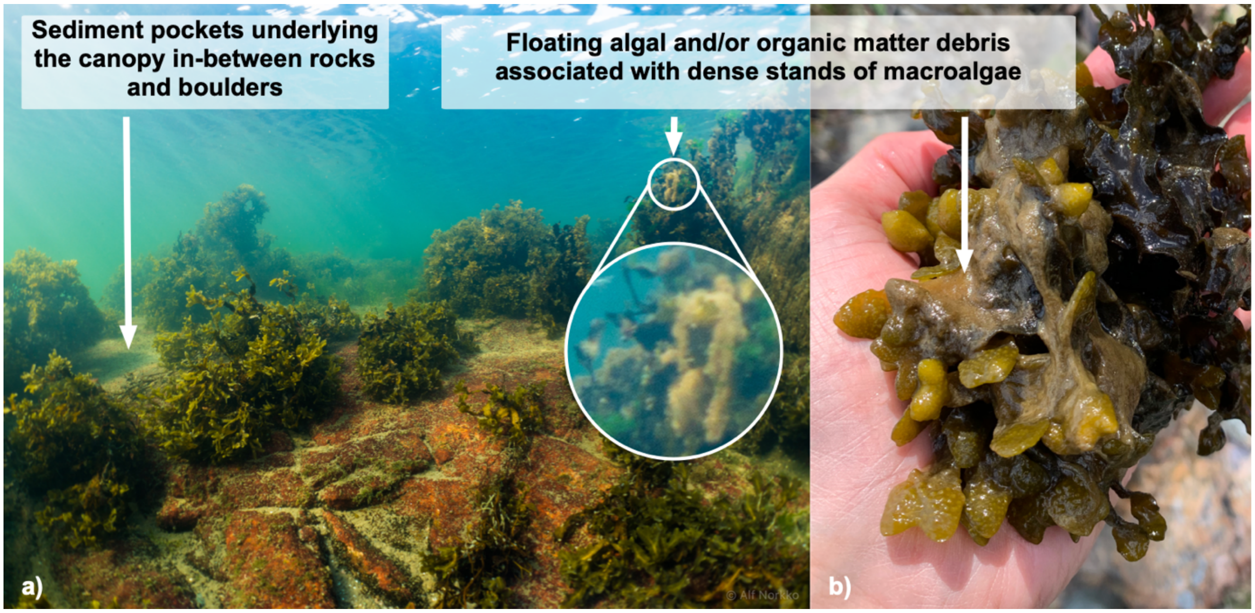


**Supplementary Figure 2**. Exemplary picture from the macroalgae habitat dominated by *Fucus vesiculosus* (A) and ex situ close-up of *Fucus vesiculosus* with associated floating algal and/or organic matter debris. Depicted in the pictures are exemplary locations from which additional samples for 16S rRNA gene amplicon sequencing were taken: i.e., sediment pockets underlying the canopy in between rocks and boulders, and floating algal and/or organic matter debris associated with dense stands of *Fucus vesiculosus*. Picture in a) taken by Alf Norkko; picture in b) taken by Florian Roth.

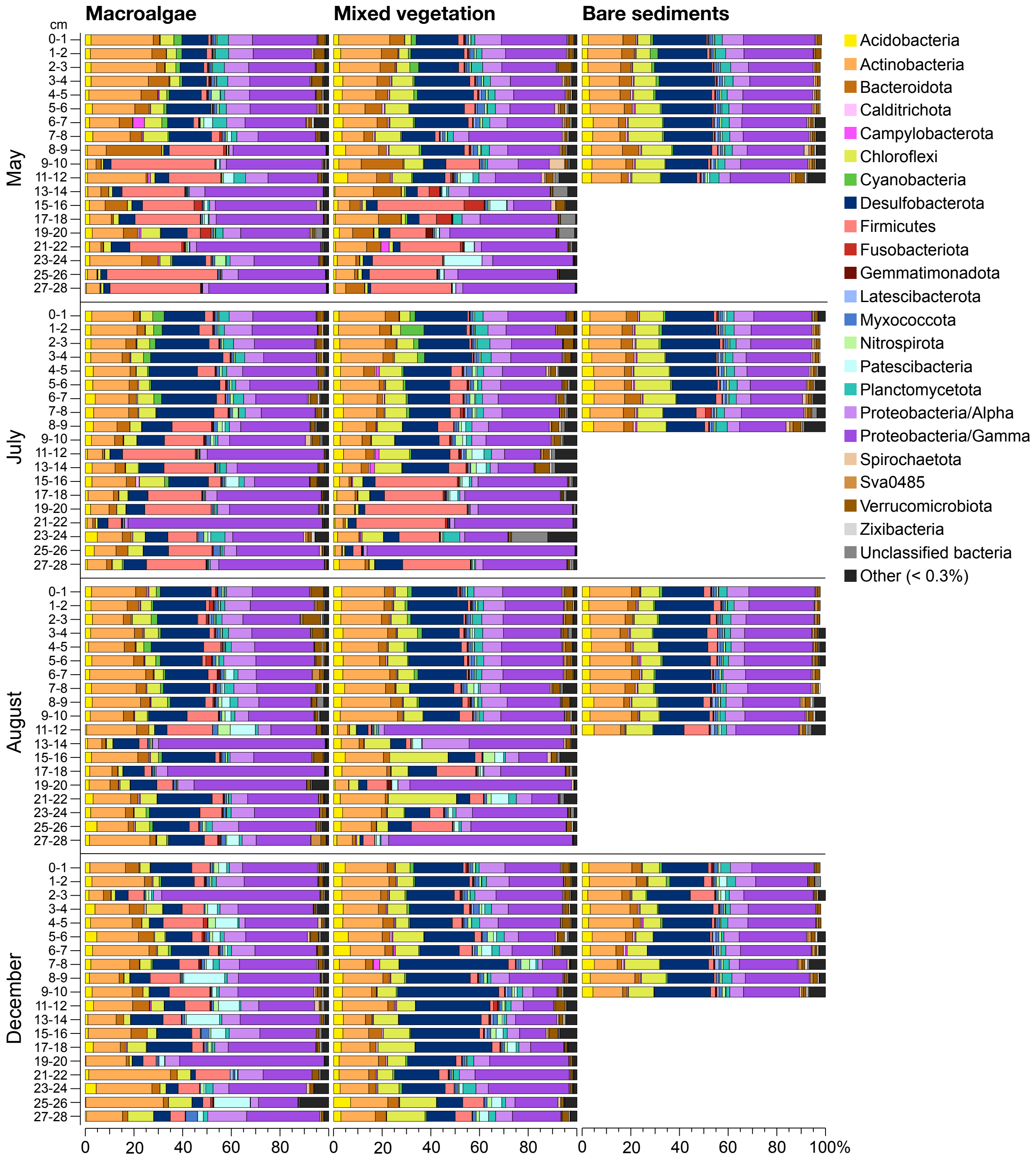


**Supplementary Figure 3.** Stacked bars showing the microbial community on the phyla level for the three habitats during sampling in May, July, August, and December. The y-axes show the different sediment layers, while the x-axes show the relative abundance (% of all phyla). "Other" denote groups < 0.3% average of all samples. Note that for the station "Sediments" samples could only be collected down to 12 cm (May), 9 cm (July), 12 cm (August), and 10 cm (December).

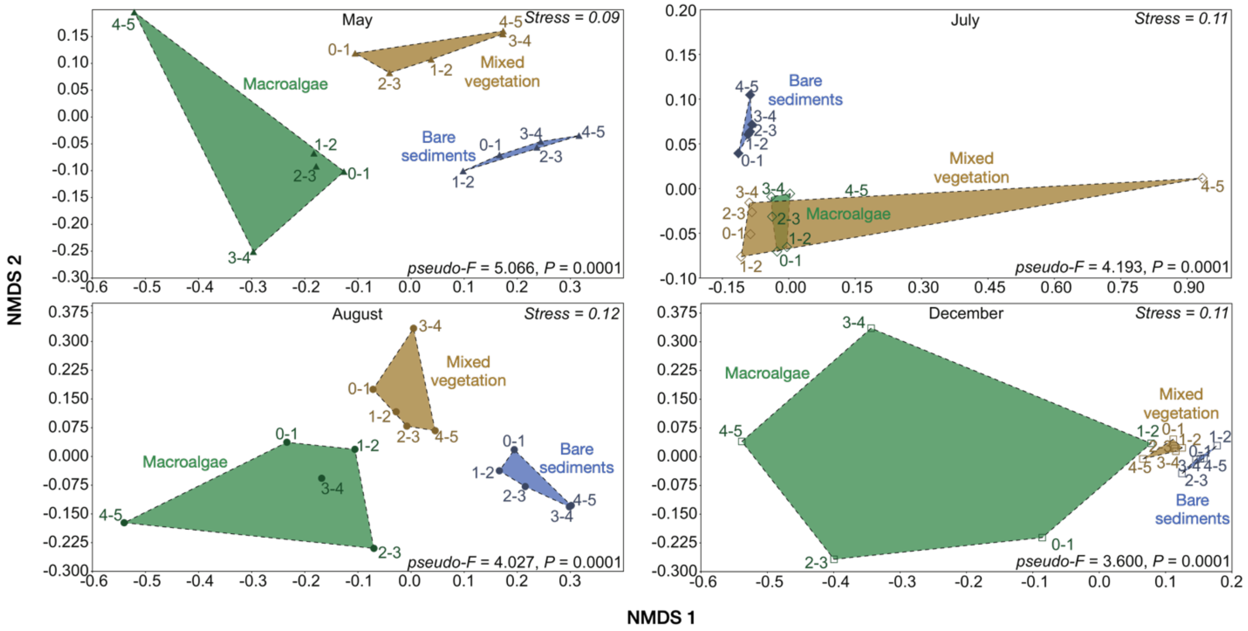


**Supplementary Figure 4.** NMDS plots of the Bray-Curtis dissimilarity index between the three habitats during sampling in May, July, August, and December. Each plot shows data from the first 5 cm layers in the sediment. The *pseudo-F* and *P* values show the statistical results from PERMANOVA (9999 permutations) based on testing all groups together.

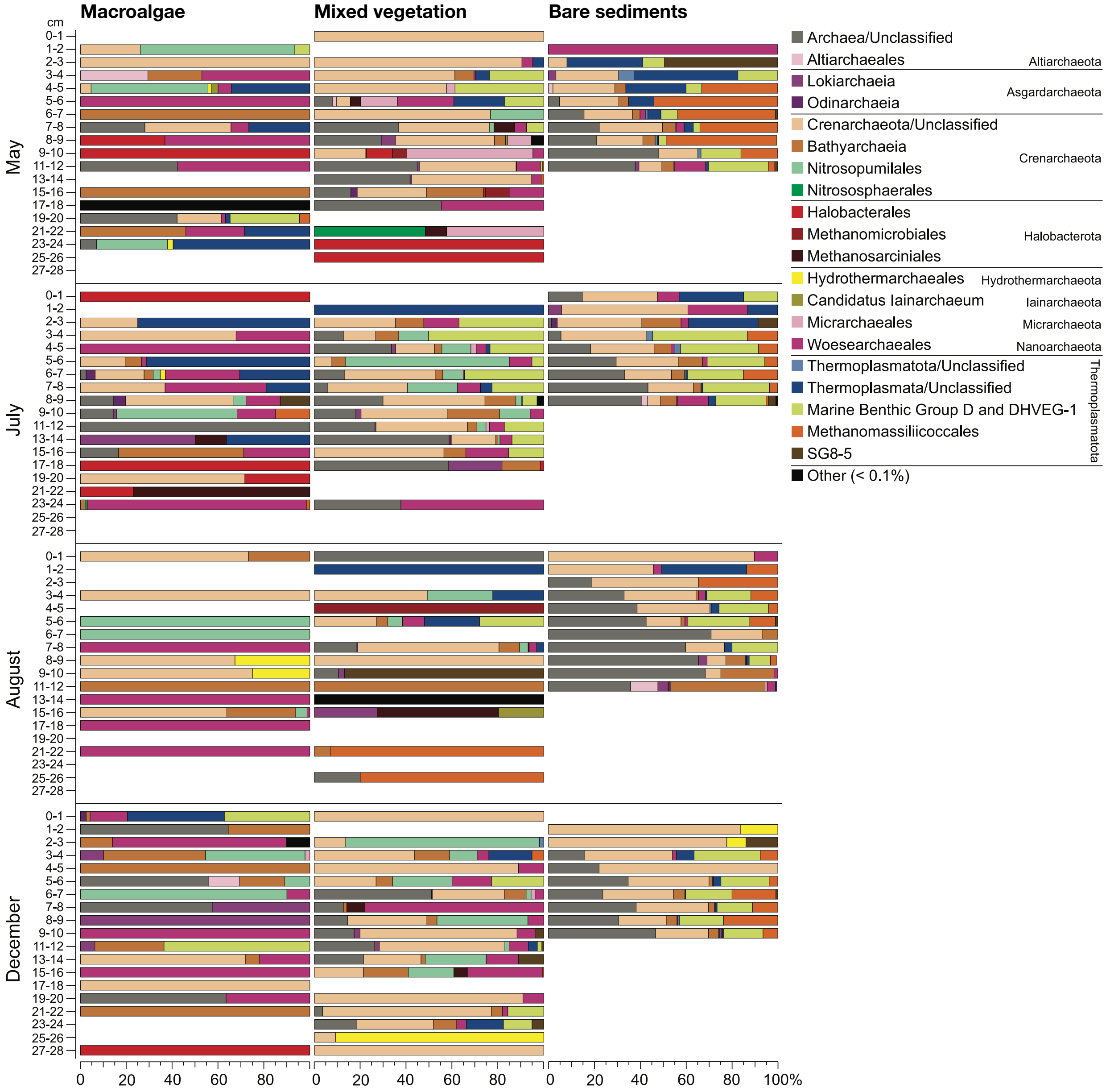


**Supplementary Figure 5.** Stacked bars showing the archaeal community on the lowest taxonomic classified level for the three habitats during sampling in May, July, August, and December. The y-axes show the different sediment layers, while the x-axes show the relative abundance (% of all archaea). "Other" denote groups < 0.1% average of all samples. Note that for the station "Sediments" samples could only be collected down to 12 cm (May), 9 cm (July), 12 cm (August), and 10 cm (December). Empty cells denote that no archaea were detected in the dataset.

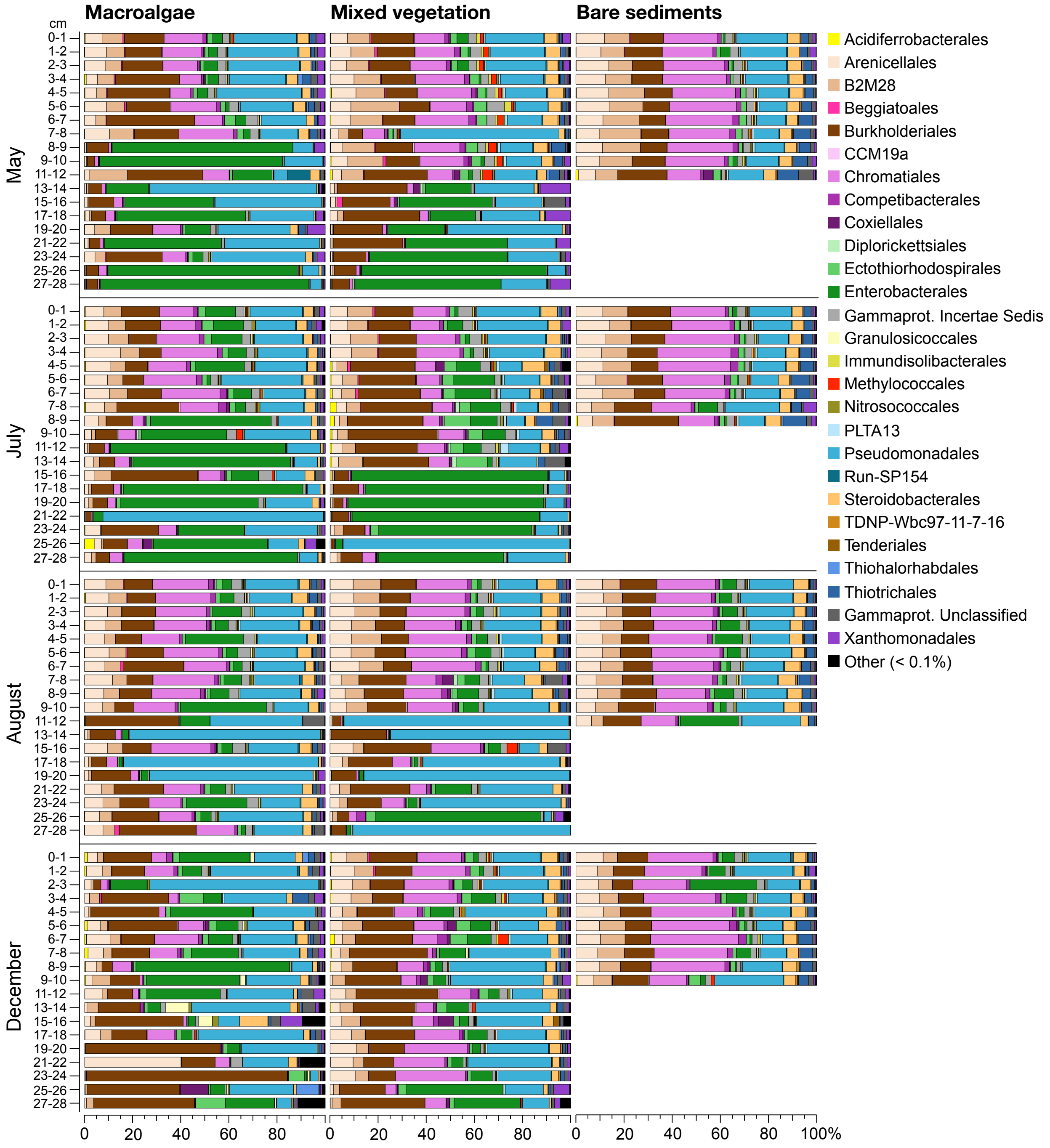


**Supplementary Figure 6.** Stacked bars showing the gammaproteobacterial community to the lowest taxonomic classified level for the three habitats during sampling in May, July, August, and December. The y-axes show the different sediment layers, while the x-axes show the relative abundance. "Other" denote groups < 0.1% average of all samples. Note that for the station "Sediments" samples could only be collected down to 12 cm (May), 9 cm (July), 12 cm (August), and 10 cm (December).

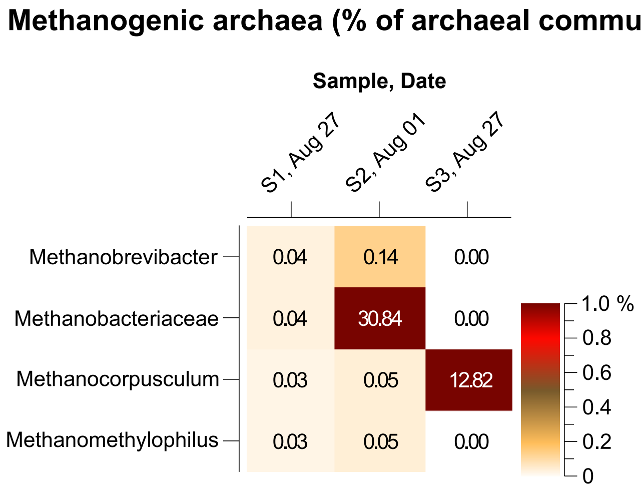


**Supplementary Figure 7.** Sequencing results from organic matter aggregates (i.e., floating filamentous algal and/or organic matter debris) associated with dense stands of *Fucus vesiculosus*. Floating filamentous algal and/or organic matter debris (exemplary pictures in Fig. S2A and S2B) were collected from within dense stands of *Fucus vesiculosus* (i.e., the macroalgae habitat) in August 2020. Shown is the relative abundance (in % of all archaea) of methanogenic archaea. The figure shows the results from sequencing of the archaeal 16S rRNA gene V4–V5 region and the lowest level of classified taxonomy.

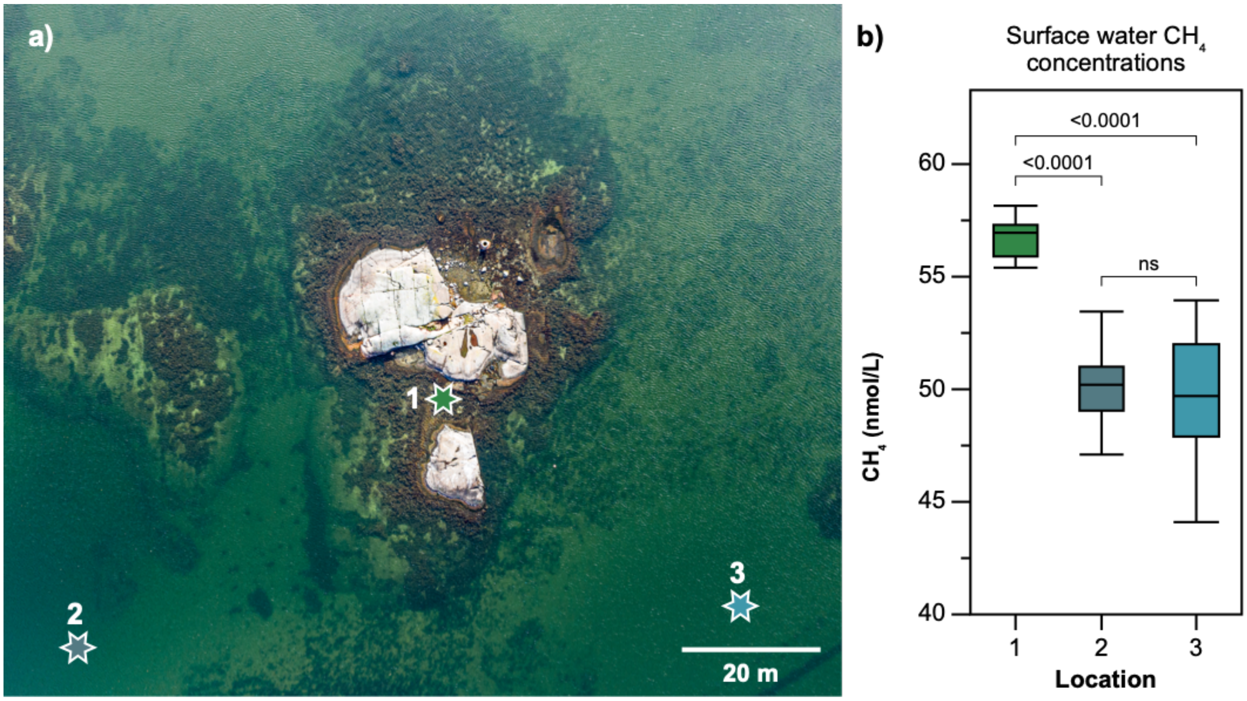


**Supplementary Figure 8.** (a) Aerial image of the sampling sites around a rocky island in the Finnish archipelago (59°50'30.8"N 23°15'01.0"E) surrounded by dense stands of *Fucus vesiculosus* (brown patches) and (b) results of surface water $CH_4$ concentration measurements at the different locations indicated in (a). Location "1" refers to within the dense macroalgae stands directly surrounding the island, while "2" and "3" are open water control sites. The concentration was recorded for approximately 1h continuously at each location between 09:00 – 12:00 on October 13, 2021. During this period, the following environmental conditions were recorded across the three stations (mean ± SD): water temperature = 11.34 ± 0.03 °C; salinity = 5.98 ± 0.00; windspeed = 3.4 ± 0.6 m/s. A one-way ANOVA was performed to compare the effect of the three different locations on surface water $CH_4$ concentrations. There was a statistically significant difference in mean surface water $CH_4$ concentrations between at least two groups ($F(2, 291) = [348.2133]$, $p < 0.0001$). Tukey's HSD Test for multiple comparisons found that the mean value of surface water $CH_4$ concentrations was significantly different between location 1 (mean = 56.69, SD = 0.76, n = 70) and 2 (mean = 50.41, SD = 1.48, n = 105) ($p < 0.0001$, 95% C.I. = [-6.95, -5.61]) and location 1 and 3 (mean = 49.73, SD = 2.48, n = 119) ($p < 0.0001$, 95% C.I. = [6.31, 7.62]). There was no statistically significant difference in mean surface water $CH_4$ concentrations between location 2 and 3 ($p = 0.167$). Abbreviations: ns = not significant. Picture in (a) taken by Alf Norkko.
