# Supplementary material for: Methane emissions offset atmospheric carbon dioxide uptake in coastal macroalgae, mixed vegetation and sediment ecosystems
Source: Nat Commun. 2023 Jan 3;14:42. doi: 10.1038/s41467-022-35673-9 (PMC9810657; doi:10.1038/s41467-022-35673-9)
Supplement: Supplementary file 3 — Description of Additional Supplementary Files [file 41467_2022_35673_MOESM3_ESM.pdf]

Description of Additional Supplementary Files:

Supplementary Data 1: Supplementary Data 1 contains a full list of sample names, fastq file names, sequences obtained before and after quality trimming, and number of amplicon sequence variants (ASVs) constructed for sediment and algal microbial data. The raw sequencing data has been uploaded to NCBI GenBank (https://www.ncbi.nlm.nih.gov/bioproj ect/) and can be accessed at BioProject PRJNA756121.
